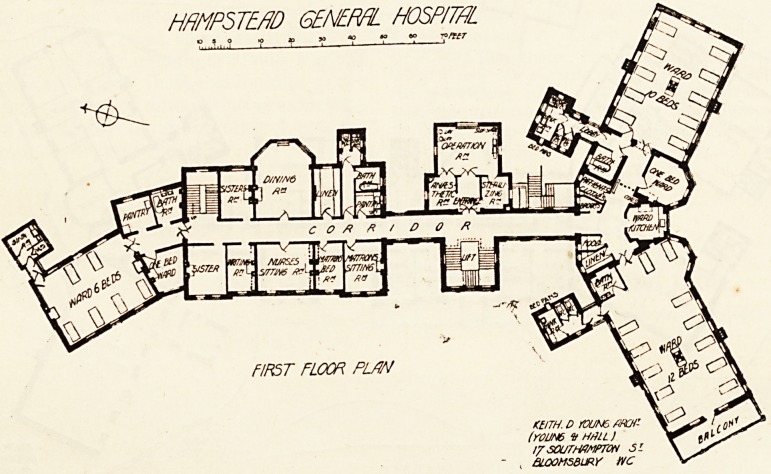# Hampstead General Hospital

**Published:** 1908-05-02

**Authors:** 


### HOSPITAL ADMINISTRATION.

### CONSTRUCTION AND ECONOMICS.

### HAMPSTEAD GENERAL HOSPITAL.

This hospital, the first portion of which was opened for patients in 1906, has now been completed, though the recently erected portions are not yet occupied.

The site is one which presents peculiar difficulties owing to its being on the side of a steep hill. The fall of the ground will be best realised from the following facts :—The ground floor of the main administration block becomes basement in the front part of the east wing of the ward block, while the back or north rooms of the latter are in their turn ground floor. The out-patient block, which is on the same level as the basement of the main building, is some three feet below the pavement on the front, and about the same distance above the ground at the back.

The building, for convenience of description, may be divided into six blocks : (1) The main administration block, having on one side the (2) pay ward block, and on the other, the (3) general ward block with the (4) staircase and operation-room block intervening between the two last-named; the (5) out-patient department, and the (6) mortuary block.

The main administration block occupies an approximately central position and is five stories in height. In the basement are the kitchen offices, servants' hall, and general store. The kitchen and scullery are large and well lighted, and communicate with each other by a large archway. The cooking apparatus consists of a large cooking range, a gas hot-closet, gas-heated steamers, and a gas grill. These and the range of sinks were all supplied by Messrs. Slater and Company.

On the ground floor in the centre is the entrance-hall, which serves also as porter's office and waiting-room. Over the mantelpiece is a beaten-copper tablet recording the fact that a sum of £1,500 for building and £226 2s. 3d. for maintenance was handed over to the hospital by the Hampstead Borough Council as a memorial of his Majesty's Coronation.

On the right of the entrance in front are staff room and house surgeon's sitting-room, and on the left matron's office and secretary's clerk's office. At the back is the board-room, having on one side the secretary's office and staircase and on the other the house surgeon's bedroom, with his bath-room, a lavatory and w.c. for staff and a pantry. On the first floor are the matron's sitting-room and bedroom, nurses' sitting-room, two sisters' rooms, nurses' dining-room, linen-room, pantry, bath-room, w.c., and housemaids' closet. The second and third floor each contain six bedrooms for nurses, a box-room, two bath-rooms, a w.c., and sink-room. The pay ward block corresponds in point of height and number of stories with the main administration block, and contains on the basement floor a room for the use of the committee of the Samaritan Fund, two rooms for $x$-ray and other electrical work, larder, room for cleaning knives and boots, and a ward for night casualty cases, with a w.c. and sink-room attached. The ground floor and the first floor each contain a ward for six beds, a one-bed ward, pantry, and bath-room, with a w.c. and sink-room in a separate tower approached by a cross-ventilated lobby. On the second floor are four one-bed wards, with pantry, bath-room, and sanitary offices all as on the floors below. The third floor contains bedrooms for eight nurses, with a bath-room and w.c.

The general ward block has no basement, and is only partly utilised on the ground-floor level. Here are eight bedrooms for servants, with two w.c.'s, a bath-room, and a

GROUND FLOOR PLAN

housemaids' closet; three store-rooms, one of which is used temporarily as dispensary and one for sterilising dressings, and two rooms for the reception of foul and soiled linen pending its despatch to the laundry. An iron shoot with doors on all floors is attached to the framework of the outside staircase, and is fed from each landing. The first floor and the two floors over are similar in all respects, and eacn contain two large wards, one for twelve and one for ten beds each, with its own bath-room and its sanitary offices in a detached tower connected with the ward by covered and cross-ventilated bridges. These towers have on each floor two w.c.'s for patients and a large sink-room, and the tower belonging to the eastern wing contains a w.c. for nurses' use entered from the sink-room. A ward kitchen, one-bed ward, linen-store, room for patients' clothes, a cupboard for brooms, etc., and one for food complete the accomomodation on each ward floor. The western wards are provided with wide balconies facing west and accessible from the ward by wide French windows. This west wing is named the "Harben Wing," after Sir Henry Harben, a

The centre part between the two wards is carried up to a fourth story, and there, approached by the open iron staircase, are two isolation wards, a nurses' room, bath-room, pantry, and sanitary offices.

The operation-room block contains the main staircase, in the well of which is a hydraulic life, with a car adapted for conveying a patient on a stretcher. This lift travels from the basement to the third floor.

The building at the back has a sub-basement, in which is the boiler-house. The basement contains the general linen-store and work-room, a large room fitted with presses and racks and warmed by hot water.

On the ground floor is the casualty department consisting of waiting-room, surgery, and examining-room. On the first floor is the operation-theatre, sterilising-room, and anæsthetic-room. The walls of all these rooms and the ceiling of the operation-room are lined with glass tiles, and the floors are laid with vitreous mosaic. The operation-room and the anæsthetic-room are warmed by means of pivoted hot-water radiators. Fresh air is forced in by means of electric fans, and the shafts for extraction are connected to munificent benefactor, to whom the existence of the hospital is largely due. It also bears a tablet recording the fact that in a house formerly occupying the site, Sir Rowland Hill, the originator of the penny post, lived for many years.

The east wards have teak floors laid direct on the concrete, while the west wards are floored with terrazzo, a plastic material which has an impervious surface which is susceptible of a high polish, and, having no joints, should prove a very valuable form of flooring for hospital purposes. All the wards in the pay ward block are floored in a similar manner, and the same material has been used throughout the basement of the pay ward block and the out-patient department. The cost (5s. to 6s. per square yard) is another element in its favour, for it is very little more than the cost of a first quality deal floor. The walls of the wards throughout are to be finished with a washable distemper.

The large wards are warmed by central Teale grates, with descending flues passing under the floors and connected to vertical shafts. On one side of each pair of grates is a recess for a water boiler, and on the other one for an instrument steriliser, each vessel being provided with a small gas stove and a flue for carrying off the fumes into the smoke flue. This arrangement was carried out by the Teale Fireplace Company from the architects' designs.

a second fan in the roof for use when required by atmospheric conditions—*e.g.*, in foggy and very damp weather.

The out-patient department is planned on a small scale, and, if the scheme of amalgamation with the North-West London Hospital is carried through, will be used only for special departments (eye, ear and throat, skin, etc.). It comprises a waiting-hall, with sanitary offices for male and female patients, inquiry office, two consulting-rooms (each with its retiring-room), a waiting-room for medicine, and the dispensary. Patients coming for medicine or dressings only can be passed through to the dispensary without going through either of the consulting-rooms, and the whole department is so arranged that no patient will retrace his or her steps or meet any other patient going in an opposite direction.

A detached mortuary and post-mortem room complete the description of the buildings.

The total cost of the buildings, including all fittings (such as lift, sterilising apparatus, etc.) and the laying out of the grounds itself—rather a heavy item, owing to the steep gradients of the roads—amounted to about £39,300, or at the rate of about £437 per bed .

The plans were prepared by Messrs. Young and Hall, under whose direction the works have been carried out.

## Figures and Tables

**Figure f1:**
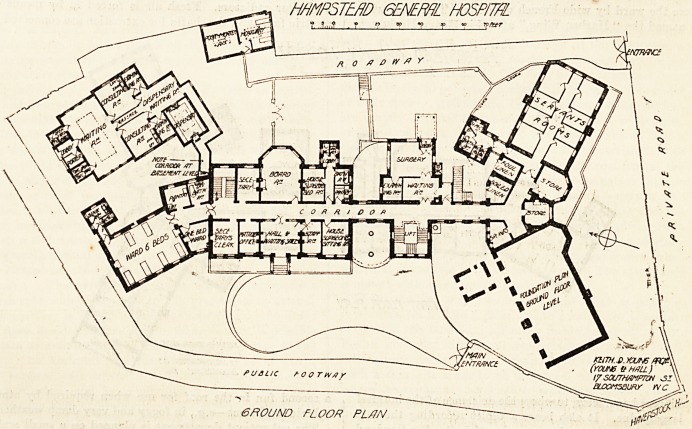


**Figure f2:**